# Plasma microRNA signatures in drug-naïve Romanian adolescents with first-episode psychosis

**DOI:** 10.3389/fpsyt.2026.1837719

**Published:** 2026-05-28

**Authors:** Ștefania-Alexandra Grosu, Elena Milanesi, Iulia Andreea Pelisenco, Radu-Mihai Paun, Maria Dobre, Mihail Eugen Hinescu

**Affiliations:** 1 Faculty of Medicine, Carol Davila University of Medicine and Pharmacy, Bucharest, Romania; 2 “Professor Doctor Alexandru Obregia” Psychiatric Clinical Hospital, Bucharest, Romania; 3 Victor Babes, National Institute of Pathology, Bucharest, Romania; 4 Aproape Clinic, Bucharest, Romania

**Keywords:** adolescents, biomarkers, drug-naive, first-episode psychosis, microRNA

## Abstract

Psychotic disorders are a group of severe psychiatric conditions with onset typically occurring in adolescence or early adulthood. Despite significant efforts to identify clinically useful candidates, no validated biomarkers for psychiatric disorders currently exist. The diagnosis of psychotic disorders is exclusively based on clinical assessment, significantly affected by individual differences and symptom overlap. Although circulating microRNAs (miRNAs) have emerged as potential peripheral biomarkers for early diagnosis and disease evolution, most studies concentrate on adult, medicated cohorts. Studies on miRNA profiles in drug-naïve adolescents with first-episode psychosis (FEP) are scarce. This study aims to identify the plasma miRNA profile in treatment-naïve Romanian adolescents with first-episode psychosis and to compare it with that of age- and sex-matched healthy controls. The plasma from 14 adolescents, seven drug-naïve FEP and seven and age-matched controls (CTRL) aged 15–18 years was collected. Psychiatric symptoms were assessed using PANSS, HAM-D, and YMRS scales. The levels of 179 miRNAs were assessed using qRT-PCR. A case-control analysis on miRNAs levels between FEP and CTRL was performed, as well as correlations with clinical measures. Twenty-one miRNAs showed significantly lower levels and two higher levels in FEP patients compared to controls. After adjustment for multiple comparisons, miR-125a-5p, miR-205-5p, miR-145-5p, miR-363-3p, and miR-23b-3p remained statistically significant (FDR<0.05). Notably, miR-125a-5p, miR-23b-3p, and miR-146a-5p levels negatively correlated with psychotic, depressive, and manic symptom severity, while miR-16-5p and miR-363-3p positively correlated with symptom scores. Comparison with previous studies indicated limited overlap, reflecting potential influences of age, treatment status, and genetic or environmental factors. This work demonstrates that Romanian treatment-naïve adolescents with first-episode psychosis had a unique circulating miRNA profile correlated with symptom severity, indicating their potential as early-stage biomarkers. The results underscore the necessity of accounting for age, treatment status, and environmental variables in the interpretation of miRNA modifications in psychotic illnesses.

## Introduction

Psychotic disorders are characterized by symptoms such as hallucinations, delusions, disorganized thinking or speech, abnormal motor behavior, and negative symptoms, with variation in symptom duration and presentation distinguishing individual nosological entities within the spectrum (1).

While less prevalent than depression or anxiety, psychotic disorders are one of the most debilitating psychiatric diagnoses, acute psychotic states having been shown to cause the highest disability across all mental disorders in the 2019 Global Burden of Disease Study (2).

A study conducted in 2017 estimated the incidence of new onset affective and nonaffective psychotic episodes to be 86 per 100,000 per year for individuals 15–29 years of age and 46 per 100,000 for the 30–59 age group (3). About 15 in 100,000 individuals subsequently receive the diagnosis of schizophrenia (4). The estimated global incidence of psychotic disorders has been calculated to be around 26.6 per 100,000 person-years, with high variability across geographical regions (5, 6).

The median age of onset of psychotic disorders has been estimated to be around 25 for schizophrenia spectrum disorders and 35 for acute and transient psychotic disorders, with a peak age at onset of 20.5 and 18.5, respectively. By the age of 18, 12.3% of patients are already diagnosed with schizophrenia spectrum disorders and 6.6% with acute and transient psychotic disorders; by the age of 25, the percentages increase to 47.8% for schizophrenia spectrum disorders and 20.6% for acute and transient psychotic disorders. The age of onset was calculated to be about one year earlier in males versus females, with males experiencing higher relapse rates and lower remission rates (7).

Schizophrenia heritability has been estimated between 60–80% in twin and family studies (8), with a high risk that is strongly enriched in first-degree relatives. Early-onset schizophrenia shows stronger familial aggregation and a more severe clinical outcome compared to adult-onset forms, consistent with evidence that genetic liability is particularly high in early neurodevelopmental presentations of the disorder (9). Although schizophrenia is highly heritable, polygenic risk scores explain only a small part of the overall risk (10). Together with environmental differences and exposures, such as stress and trauma exposure, social development, and even prenatal environment, it could explain why many genetically related family members do not develop the disorder. To date, there are no established biomarkers for any psychiatric disorder, although substantial effort has been made to identify clinically meaningful candidates. Psychotic disorders are currently diagnosed exclusively based on clinical assessment, which is substantially affected by interindividual heterogeneity and by symptom overlap. Moreover, early stages of the disorder may often present with ambiguous symptomatology, and diagnosis is especially difficult to be established in children and adolescent patients due to the lack of specificity (11).

The identification of objective biomarkers for first-episode psychosis could help stratify patients by risk of conversion to schizophrenia, predict treatment response, or adverse effects. Additionally, markers identified at disease onset are not affected by treatment exposure or chronicity of the disease, and therefore could provide valuable insight into the pathophysiology of psychosis (12).

While multiple studies are investigating central nervous system biomarkers, they have the disadvantages of being expensive and technically demanding, issues that may be solved by identifying peripheral biomarkers (13, 14).

Biomarkers derived from peripheral blood are easily accessible, and microRNAs (miRNAs) represent a promising class, being relatively stable to degradation by enzymes or pH variations, and could offer insight into the pathophysiology of the disease (15).

miRNAs are small non-coding RNA molecules, ranging between 21 and 25 nucleotides in length, that play a critical role in the post-transcriptional regulation of gene expression by binding to messenger RNA (mRNA) and either inhibiting gene translation or causing mRNA degradation. They are present in peripheral blood both intracellularly and extracellularly, allowing their detection in various types of samples, such as whole blood, plasma, serum, peripheral blood mononuclear cells or extracellular vesicles (16–19).

Although a substantial number of first episode psychosis (FEP) diagnoses are made before the patient is 18 years old (7), there are very few studies that include children and adolescents to investigate miRNAs profile in psychotic disorders. Current literature suggests that miRNA levels vary considerably with age (20), therefore the wide age ranges that are currently used in the inclusion criteria of case-control studies may not be suitable for miRNA profiling.

A systematic review published in 2023 indicated that there are several miRNAs that have been consistently found to be dysregulated in patient samples compared to controls, notably miR-34a, miR-181b, miR-432, miR-30e (21). It is worth mentioning that most of the patients included in these studies were adults and many had been previously diagnosed with schizophrenia and received pharmacological treatment.

Research investigating the role of microRNAs in psychotic diseases in Eastern European populations is relatively scarce and concentrated within a small number of research institutions, such as Poland (22) and Belarus (23).

Despite these contributions, the existing studies have concentrated on adult schizophrenia populations, and research specifically targeting treatment-naïve adolescents or the early stages of psychosis in Eastern European populations is largely lacking, and new studies in this direction are needed.

Therefore, the aim of this study is to characterize the circulating miRNA profile in treatment-naive Romanian adolescents diagnosed with first episode psychosis, and comparing this profile with those of an age- and sex-matched healthy control group.

## Materials and methods

### Individuals and sample collection

A total of 14 adolescents were included in this study: 7 inpatients diagnosed with first episode psychosis, admitted in the “Prof. Dr. Alexandru Obregia” Clinical Hospital of Psychiatry in Bucharest, Romania, and 7 age- and sex- matched controls. The samples were collected between March 2024 and October 2025. The inclusion criteria for the FEP group were the following: i) A diagnosis of Acute and Transient Psychotic Disorder based on the International Statistical Classification of Diseases and Related Health Problems 10^th^ Revision (ICD-10) (24). ii) No previous psychiatric diagnoses. iii) No history of psychotropic medication in the previous 3 months, or no more than 7 days of treatment in the current episode. iv). Ages between 15–18 years. Participants were deemed ineligible for inclusion if they met any of the following exclusion criteria: i). Use of psychotropic medication within 7 days prior to blood collection or at any time within the preceding 3 months; ii). Prior diagnosis of any mental illness; iii) Any organic diagnoses that could cause psychotic symptoms, such as head trauma, epilepsy, encephalitis, or other diagnoses based on clinician assessment; iv) Any documented premorbid developmental abnormalities or intellectual disability; v) Substance use (including alcohol, illicit drugs); vi). The refusal of consent from the patient or guardian.

Informed consent was obtained from all patients and controls and their legal guardians, and the study was approved by the hospital’s ethics committee (No. 137/22.02.2024) and was conducted in accordance with the Declaration of Helsinki. Peripheral venous blood was collected in EDTA tubes for each participant and immediately processed (up to 1 hour) for plasma isolation, using a two-step centrifugation protocol: 1900 × g for 10 minutes at 4 °C to separate the plasma, followed by centrifugation at 16,000 × g for 10 minutes at 4 °C to eliminate any remaining cellular debris. The resulting plasma was stored at −80 °C until miRNA isolation and purification.

### Psychiatric assessment

The psychosis and affective dimensions were assessed using standardized clinical rating scales. Psychotic symptoms were evaluated using the Positive and Negative Syndrome Scale (PANSS) for psychosis (25), which comprises 30 items divided into 3 subscales: the Positive subscale (7 items), evaluating symptoms such as delusions, hallucinations, or disorganization; the Negative subscale (7 items), which measures symptoms such as blunted affect or emotional withdrawal; and the General Psychopathology (16 items) that includes the assessment of a broad range of symptoms, including anxiety, guilt, or cognitive impairment. Each item is rated on a scale from 1 (absent symptom) to 7 (extreme), higher scores indicating greater symptom severity, and the subscales allow differentiation between symptom domains.

The Hamilton Depression Scale (HAM-D) was used for depressive symptoms. HAM-D is a widely used instrument, with its most commonly used version comprising 17 items, each rated on scales from either 0 (absent symptom) to 4 (extreme), or from 0 to 3. The total scores range from 0 to 52, with four degrees of symptom severity: 0-7 (no depression); 8-16 (mild depression); 17-23 (moderate depression); and 24-52 (severe depression). The scale includes the assessment of various symptoms such as: mood, guilt, anxiety, suicidal ideation, sleep disturbances, psychomotor changes, and somatic symptoms (26). Manic symptoms were assessed using the Young Mania Rating Scale (YMRS). This scale includes 11 items rated on a scale from either 0–4 or 0-8, depending on the symptom, with a maximum score of 60. YMRS evaluates symptoms such as: elevated mood, increased motor activity/energy, sexual interest, sleep, irritability, speech, language/thought disorder, disruptive/aggressive behavior, appearance, or insight. Symptom severity is staged as follows: 0–12 = euthymic/normal, 13–19 = mild mania, 20–25 = moderate mania, and ≥26 = severe mania (27).

### miRNA expression analysis

Following the manufacturer’s protocol, RNA was isolated from 200 µL of plasma using the miRNeasy Serum/Plasma Mini Kit (Qiagen, Hilden, Germany) and was eluted in 20 µL of nuclease-free water. Six µL of RNA was used for reverse transcription with the miRCURY LNA RT Kit (Qiagen, Hilden, Germany). The level of miRNAs included in the validated circulating miRNA array was assessed using the Human Serum/Plasma Focus miRCURY LNA miRNA Focus PCR Panel YAHS-406Z (Qiagen, Hilden, Germany) via qRT-PCR. Amplification was performed using SYBR Green chemistry on an AriaMx Real-Time PCR System (Agilent Technologies, Santa Clara, CA, USA). Ct values were normalized against the geometric mean of the two most stable miRNAs let-7b-5p and miR-101-3p. The stability was established using the RefFinder algorithm (28) based on the 67 most abundantly expressed miRNAs (Ct value< 30) out the 179 miRNAs available in the panel. The levels of miRNAs are presented as fold regulation (FR) and as 2^−ΔCt^ values.

### Statistical analysis

The Statistical Package for Social Sciences (SPSS version 20.0) was used for statistical analysis. Continuous variables were tested for normality using the Shapiro–Wilk test. As the sample size was relatively small and most continuous variables did not follow a normal distribution, non-parametric tests were applied.

The Mann–Whitney U test was used to compare sociodemographic and clinical characteristics, as well as plasma miRNA expression levels between FEP adolescents and controls. Categorical variables were compared using the Chi-square test or Fisher’s exact test, as appropriate. The levels of miRNAs were calculated using the 2^−ΔCt^ method. P-values were adjusted for multiple comparisons using the Benjamini–Hochberg procedure to control the false discovery rate (FDR). Statistical significance was defined as FDR< 0.05. When applicable, correlations between miRNA levels and clinical variables were assessed using Spearman’s rank correlation coefficient.

To assess the robustness of the findings, effect sizes were calculated for each significant miRNA to quantify the magnitude and direction of group differences. Given the non-normal distribution of 2^-ΔCt^ values, Cliff’s delta (δ), a non-parametric effect size measure, was used. Effect sizes were interpreted using established benchmarks for rank-based dominance measures (29, 30) as follows: |δ| < |0.147| were considered negligible, indicating substantial overlap between groups. Values | 0.147≤ |δ| < 0.330| were interpreted as small effects, suggesting limited separation. Values | 0.330≤ |δ| < 0.474| represented medium effects, reflecting moderate group separation. Values of |δ| ≥ |0.474| were considered large effects, indicating strong separation between groups, while values approaching ±1 indicated near-complete distributional separation. The Cliff’s delta (δ) for each analyzed miRNA was reported in **Table 1** next to the p-value obtained using the Mann-Whitney U test.

**Table 1:** MicroRNAs showing significantly different levels between FEP and CTRL.

miRNA	FR (FEP vs CTRL)	P-value^a^	Cliff’s δ	Adj p-value
miR-125a-5p	-3.79	0.001	-1	0.04
miR-338-3p	-2.92	0.026	-0.71	0.156
miR-1260a	-2.44	0.007	-0.83	0.084
miR-23b-3p	-2.34	0.002	-0.92	0.048
miR-221-3p	-2.24	0.011	-0.80	0.094
miR-27a-3p	-2.12	0.011	-0.80	0.094
miR-30a-5p	-2.09	0.004	-0.88	0.06
miR-205-5p	-2.02	0.001	-0.96	0.04
miR-92b-3p	-1.99	0.026	-0.71	0.156
miR-197-3p	-1.99	0.004	-0.88	0.06
miR-146a-5p	-1.99	0.038	-0.67	0.198
miR-23a-3p	-1.82	0.007	-0.83	0.084
miR-27b-3p	-1.80	0.038	-0.67	0.198
miR-125b-5p	-1.78	0.004	-0.88	0.06
miR-145-5p	-1.69	0.001	-0.96	0.04
let-7d-5p	-1.64	0.011	-0.80	0.094
miR-26a-5p	-1.63	0.026	-0.71	0.156
miR-301a-3p	-1.54	0.017	-0.75	0.127
miR-29c-3p	-1.42	0.017	-0.75	0.127
let-7e-5p	-1.34	0.011	-0.80	0.094
miR-191-5p	-1.31	0.038	-0.67	0.198
miR-16-5p	1.24	0.026	0.787	0.156
miR-363-3p	1.52	0.002	0.92	0.048

^a^ Mann-Whitney U test.

## Results

### Patients and controls characteristics

The study included seven drug-naïve adolescents with FEP patients and seven controls (CTRL), matched according to age, sex, ethnicity, occupation, and lifestyle factors (all p > 0.05). Sleep duration was slightly more variable among FEP participants but did not differ significantly from that of controls (p = 0.192). Only one patient reported a first-degree relative with a psychiatric disorder.

FEP participants exhibited significantly higher symptom severity across all clinical measures. PANSS total and subscale scores were elevated in FEP compared to controls, and HDRS and YMRS scores were also higher in the FEP group (**Table 2**).

**Table 2:** Demographic and clinical characteristics of patients with first-episode psychosis and controls.

Characteristics	FEP (n=7)	CTRL (n=7)	P-value
Age (Mean ± SD)	17.14 ± 1.95	15.57 ± 1.51	0.209^a^
Sex (%F)	57.14%	57.14%	1.000^b^
Ethnicity	100% Romanian	85.7% (n=6) Romanian 14.3% (n=1) Roma	1.000^b^
Occupation	100% Student	85.7% (n=6) Student 14.3% (n=1) NEET	1.000^b^
Smokers	100% No	100% No	1.000^b^
Alcohol consumers	100% No	100% No	1.000^b^
Coffee consumers	100% No	85.7% (n=6) No 14.3% (n=1) Yes	1.000^b^
Sleeping hours	<5 hours (n=1) 5–6 hours (n=2) 6–7 hours (n=4)	6–7 hours (n=7)	0.192^b^
PANSS Total (Mean ± SD)	101.71 ± 35.720	41.00 ± 8.103	0.001^a^
PANSS Positive (Mean ± SD)	24.14 ± 5.047	8.14 ± 1.215	0.001^a^
PANSS Negative (Mean ± SD)	24.57 ± 12.026	11.57 ± 3.910	0.001^a^
PANSS General (Mean ± SD)	53.00 ± 20.672	21.29 ± 4.821	0.001^a^
HDRS (Mean ± SD)	10.57 ± 4.077	2.00 ± 1.528	0.001^a^

aMann-Whitney U test; bFisher’s Exact Test; NEET, not in education, employment, or training.

### Plasma miRNA levels and correlations with clinical features

**Figure 1:**
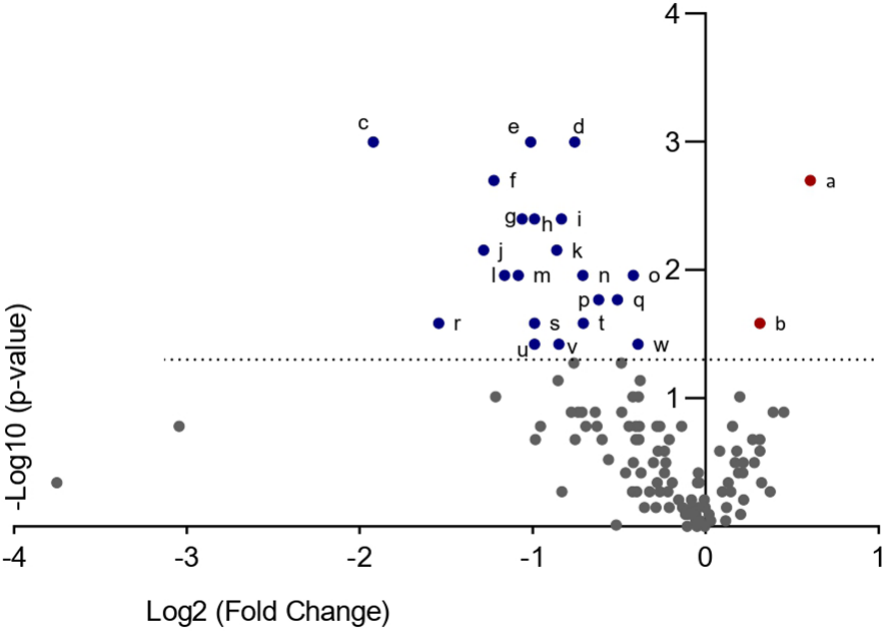
Volcano plot showing differential expression of miRNAs between patients with first-episode psychosis (FEP) and controls, as measured by qRT-PCR. The x-axis represents log_2_ fold change (FEP vs. CTRL), and the y-axis represents −log_1__0_(p-value) derived from the Mann–Whitney U test. Each point corresponds to an individual miRNA. miRNAs meeting the significance threshold (p < 0.05) are highlighted, indicating significantly upregulated (red) and downregulated (blue) miRNAs in FEP compared to CTRL.

Legend: (a) miR-363-3p; (b) miR-16-5p; (c) miR-125a-5p; (d) miR-205-5p; (e) miR-145-5p; (f) miR-23b-3p; (g) miR-30a-5p; (h) miR-197-3p; (i) miR-125b-5p; (j) miR-1260a; (k) miR-23a-3p; (l) miR-221-3p; (m) miR-27a-3p; (n) let-7d-5p; (o) let-7e-5p; (p) miR-301a-3p; (q) miR-29c-3p; (r) miR-338-3p; (s) miR-92b-3p; (t) miR-26a-5p; (u) miR-146a-5p; (v) miR-27b-3p; (w) miR-191-5p.

In the initial investigation, the plasma miRNA levels of the FEP and control groups were compared. Using a significance threshold of p < 0.05, we identified 21 microRNAs with significantly lower levels and two microRNAs with significantly higher levels in FEP individuals compared to controls (**Table 1**). Data are visualized in a Volcano Plot reported in **Figure 1**. After adjustment for multiple comparisons, miR-125a-5p, miR-205-5p, miR-145-5p, miR-363-3p, and miR-23b-3p remained statistically significant (FDR<0.05).

**Figure 2:**
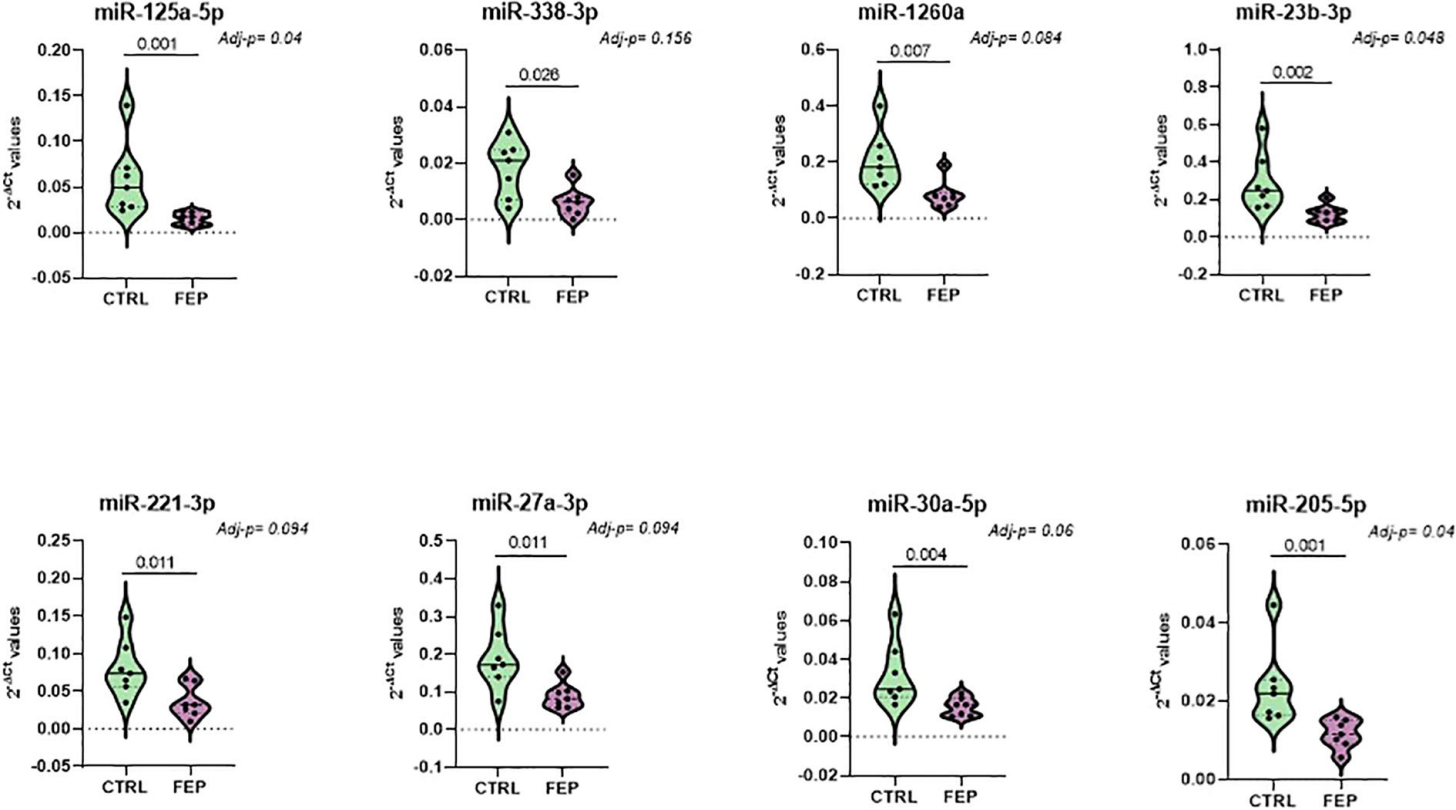
Violin plots showing the distribution of individual 2^-ΔCt^ values for each group. Each dot represents a single individual sample. The solid horizontal line indicates the median value, while the dotted horizontal lines represent the interquartile range (25th and 75th percentiles). The width of the violin corresponds to the kernel density estimation of the data distribution. Only miRNAs exhibiting a fold regulation of < −2 in the case–control comparison are shown. p-values were calculated using the Mann–Whitney U test and adjusted for multiple comparisons using the Benjamini–Hochberg false discovery rate (FDR) procedure.

After applying a fold regulation (FR) filter (considering only FR values >2 or <−2 as significant), eight microRNAs were considered for the graphical representation (**Figure 2**). Among these three miRNAs were significant also after adjustment for multiple comparisons (miR-125a-5p, miR-205-5p, and miR-23b-3p).

**Figure 3:**
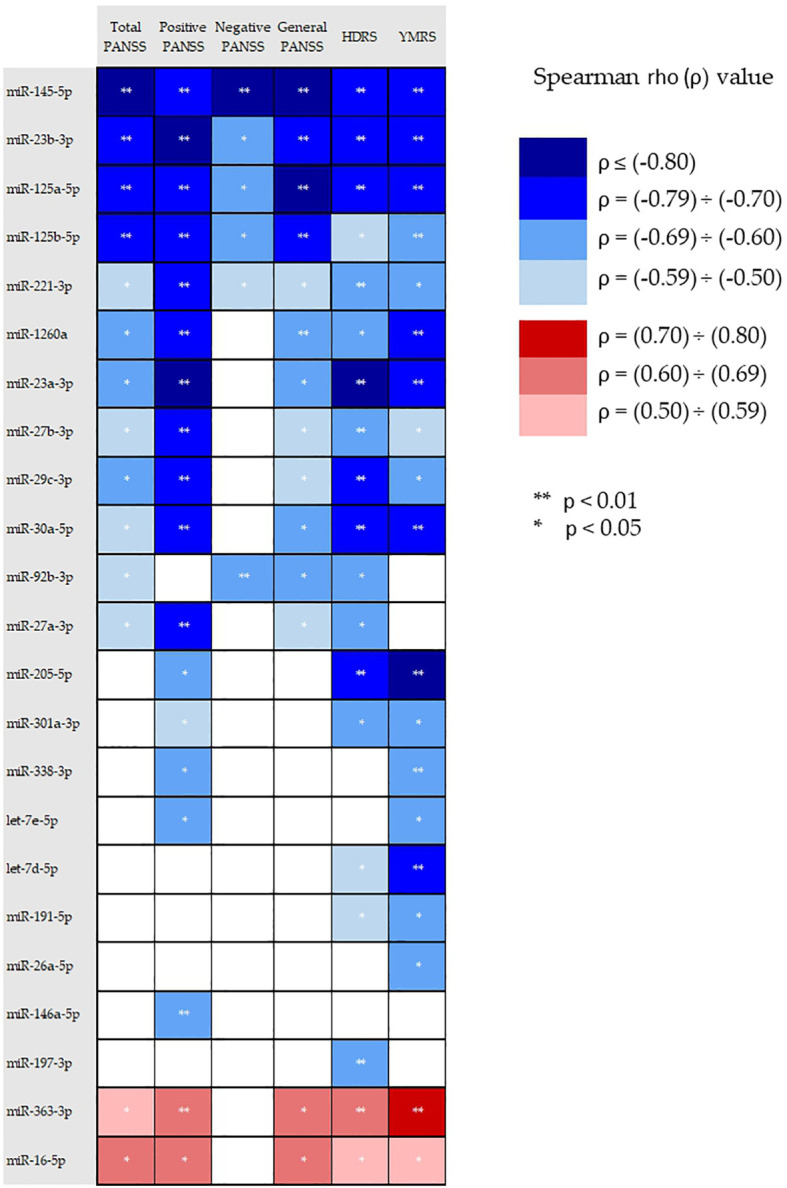
Heatmap showing correlations between the level of miRNAs and psychiatric scale scores in the entire cohort (FEP and CTRL). Correlations were calculated between the 2^-ΔCt^ values of miRNAs reported as significantly altered in **Table 1** and scores from the clinical scales. Blue shading indicates negative correlations, and red shading indicates positive correlations, with color intensity reflecting the strength of the correlation. Statistical significance is indicated by asterisks (*p < 0.05; **p < 0.01, Spearman correlation).

Correlation analysis between the levels of circulating miRNAs and psychiatric scale scores was performed considering the entire cohort of 14 adolescents. The two miRNAs that were elevated in FEP patients (miR-16-5p and miR-363-3p) showed a positive correlation with psychiatric scale scores, indicating that higher levels of these miRNAs are associated with more severe symptoms. In contrast, miR-145-5p, miR-23b-3p, miR-125a-5p, miR-125b-5p, and miR-221-3p exhibited significant negative correlations with all assessed scales, including Total PANSS, Positive PANSS, Negative PANSS, General PANSS, HDRS, and YMRS, suggesting that these miRNAs may serve as potential markers for multiple symptom domains associated with psychotic disorders (**Figure 3****).**

The results concerning the miRNAs identified as having different levels in the FEP vs CTRL analysis were compared with findings reported in the literature on psychotic and other psychiatric disorders (**Supplementary Table 1**).

## Discussion

The present study aimed to identify the circulating miRNA profile in a group of Romanian drug-naïve adolescents diagnosed with first-episode psychosis.

The results indicate the presence of several differently dysregulated miRNAs in the plasma of FEP patients compared to healthy controls. One interesting aspect of the results is that all but two of the statistically significant differences identified had the same trend- that is, the downregulation of miRNAs.

Specifically, 21 miRNAs displayed lower levels in the FEP group compared to healthy controls, while two miRNAs were more abundant in the patient group. When adjusting for multiple comparisons, miR-125a-5p, miR-205-5p, miR-145-5p, miR-363-3p, and miR-23b-3p remained statistically significant (FDR<0.05). Among the most notable findings, miR-125a-5p had levels approximately fourfold lower in the FEP group and was significantly negatively correlated with symptom severity in psychotic disorders, as well as with manic and depressive symptoms. Literature data identified this miRNA to be dysregulated in the plasma of both major depressive disorder and bipolar affective disorder, although, as opposed to the present study, miR-125a-5p was found to be upregulated in both groups compared to controls. However, as reported by the authors, the results should be interpreted carefully since the lack of data on drug exposure and the difference in age between patients and controls may confound the observed alterations in miRNAs levels (31). The same miRNA has been found to be downregulated in lymphoblastoid cell lines isolated from lithium responsive bipolar patients compared to non-responders (32). A recent study has also associated the upregulation of miR-125a-5p with late-onset Alzheimer’s disease, but there is currently no evidence of its dysregulation in psychotic disorders. Therefore, its involvement in neuropsychiatric conditions remains unestablished (33).

Comparisons of our results with existing literature identified a partial overlap of a small subset of miRNAs, with three other miRNAs (miR-23a-3p, miR-26a-5p, and miR-146a-5p) found to be dysregulated in psychotic disorders. These studies reported inconsistent results. For example, in line with our results, miR-23-a-3p was found to be downregulated in extracellular vesicles-depleted plasma of patients with schizophrenia compared to controls (34), as well as in the peripheral blood of schizophrenia patients recruited before any antipsychotic treatment or in the absence of psychotropic medication for at least three months prior to the study (35).

In contrast to our study, research conducted on the Iranian Azeri population, including patients with recent-onset non-affective disorder, suggested that increased serum levels of miR-26a-5p could be a suggestive biomarker for schizophrenia in the early stage (36). The same results have been obtained by Du and collaborators, who identified higher levels of miR-26a-5p in the plasma exosomes of ten first-episode schizophrenia patients compared to age-matched controls. Similarly, not in line with the results of the present article, higher levels of miR-146a-5p in the same group of patients were identified (37).

Only two studies have examined miRNA profile in treatment-naïve FEP population, whose age range is partially analogous to that of the adolescents in our cohort. Nonetheless, our population was generally younger and more homogeneous.

Zhao et al (38) analyzed the plasma miRNA profiles of 31 patients of Japanese origin aged between 15-40, diagnosed with either first episode schizophrenia or schizophrenia, who were treatment naive, and compared them to an age and sex matched control group. Global miRNA expression was profiled by microarray, and qRT-PCR was used for subsequent validation. Within the first episode schizophrenia group, miR-223-3p and miR-6131 were found to be significantly upregulated compared to controls. After including the schizophrenia group, only miR-223-3p remained significantly overexpressed in psychiatric patients.

The second study, conducted in Brazil (39) investigated the miRNA expression profile of 12 treatment-naive patients diagnosed with first episode psychosis (mean age 27.75 ± 7.9), selected from a larger cohort aged between 16 and 35 years (40), compared to 12 age-matched controls. RNA was isolated and sequenced from small extracellular vesicles, and 2 miRNAs were found to be downregulated compared to the control group: miR-335-5p and miR-30d-5p. Despite the similarity between the samples investigated in these two studies and the one included in the current article, no overlap between significantly dysregulated miRNAs could be found, indicating that there might be great genetic and epigenetic interregional variability.

A key strength of our study is the robust inverse correlation between miRNA levels and clinical symptom severity across psychotic and affective domains. It is worth mentioning that some miRNAs that scored strong correlations with affective symptoms were also found in other studies to be correlated with either major depressive disorder or bipolar affective disorder. Of those, miR-1260a and let-7e-5p were negatively correlated with mania, and miR-301a-3p was negatively correlated with depression, corresponding to the results of other similar studies (38, 41, 42).

The present study is, to our knowledge, the first focused on a Romanian adolescent FEP population and one of the few examining drug-naïve individuals at this early disease stage. The decision to include a very narrow age group was taken in order to eliminate the effect that aging and neurodevelopment have been proven to have on circulating miRNA expression. Notably, multiple molecules of the miRNAs described above were found by previous studies to be involved in brain development (43–45).

Additionally, identifying the miRNA profile in early psychosis and in patients minimally affected by medication could allow for a clearer characterization of molecular alterations in this stage of the disorder, using peripheral biomarkers that are easy to obtain and to analyze. The inclusion of psychiatric scales could provide insight into possible transdiagnostic markers of mental illness.

Summarizing our results, very few miRNAs identified in this study have been reported by other similar studies (21). The differences between our results and those published in earlier research could be explained by a number of factors. The variations in study populations and clinical features are important factors to take into account. In contrast to our cohort, which was exclusively made up of drug-naïve teenagers going through their first episode of psychosis, the majority of previous research has been done on adult patients, many of whom were undergoing antipsychotic treatment at the time of sampling. Direct comparisons are difficult because circulating miRNA profiles are known to be significantly influenced by both age-related biological variation and medication exposure. Furthermore, no comparable research has ever been done in Romanian psychotic populations, and larger studies frequently use more diverse samples and seldom concentrate on people under the age of 18.

The observed discrepancies are probably also influenced by geographic and environmental factors. An increasing amount of data suggests that external exposures like air pollution, cigarette smoke, nanoparticles, and other chemical agents can affect miRNA expression. For instance, human studies have consistently linked smoking to altered expression of miR-146a (46). These factors may differ significantly between populations and geographical areas, which further restricts the reproducibility of results.

More generally, Western, educated, industrialized, wealthy, and democratic (WEIRD) populations have been the focus of most psychiatric research (47), raising questions about the generalizability of findings. The biological foundations of psychotic disorders may be influenced by variations in cultural context, socioeconomic circumstances, lifestyle, environmental exposures, and healthcare systems. For a more thorough understanding of disease mechanisms, research in underrepresented groups is still crucial, and care should be taken when extrapolating results across diverse populations.

Identifying biomarkers of psychiatric disorders could aid in early diagnosis, prognosis of disease progression, and response to treatment, developing personalized medicine, and possibly reestablishing diagnostic entities based on objective, biological data. Future studies in this field should focus on validating existing results in larger cohorts that maintain homogeneity, include the current data in larger multi-omics studies, integrating genomic, epigenomic, and proteomic data, and also conduct longitudinal analysis of these parameters, which could provide insight into issues such as treatment response, relapse, and the chronic effects of psychiatric disorders.

Some limitations must be considered in our study. First, although participants were carefully selected, clinically well-characterized, and drug-naïve at the time of blood recruitment, the sample size is small. This limits the generalizability of the findings and the statistical power. Since these findings are exploratory and hypothesis-generating, rather than confirmatory, they should be interpreted with caution. We addressed these constraints using a non-parametric effect size measure (Cliff’s delta) in addition to significance testing, which is more robust in small samples and non-normally distributed data. However, independent validation on a larger, well-powered, and longitudinal cohort is warranted to validate and expand these findings in the Romanian adolescents. Secondly, the hypothesis is based on the current psychiatric nosology, which separates psychotic and affective disorders. Recent consortia biomarker studies point towards a possible common schizo-bipolar spectrum, indicating that the current distinction between the two diagnostic entities may not be biologically accurate (48).

## Conclusions

This work demonstrates that, relative to healthy controls, treatment-naïve Romanian adolescents experiencing first-episode psychosis exhibit a distinct circulating miRNA profile. The intensity of psychotic, depressive, and manic symptoms exhibited a strong correlation with several dysregulated miRNAs, such as miR-125a-5p, miR-23b-3p, and miR-146a-5p, suggesting their potential as early-stage biomarkers. These findings contribute to the increasing evidence that miRNAs may serve as objective, readily available instruments for risk assessment, early diagnosis, and monitoring of psychotic illnesses in adolescents.

## Data Availability

The raw data supporting the conclusions of this article will be made available by the authors, without undue reservation.
