# Supplementary material for: Plasma microRNA signatures in drug-naïve Romanian adolescents with first-episode psychosis
Source: Front Psychiatry. 2026 May 28;17:1837719. doi: 10.3389/fpsyt.2026.1837719 (PMC13253805; doi:10.3389/fpsyt.2026.1837719)
Supplement: Supplementary file 1 [file Table1.docx]

**Supplementary Table 1:** miRNAs with significantly altered levels in the drug-naïve adolescents Romanian FEP patients and their association with psychotic and other psychiatric disorders reported in the literature

| miRNA | Our results  FEP vs CTRL | Psychiatric disease and evidence | Biological sample | Author/year |
| --- | --- | --- | --- | --- |
| miR-146a-5p | ↓ | Schizophrenia ↑ | Plasma exosomes | Du et al. 2023 |
| miR-23a-3p | ↓ | Schizophrenia ↓  Schizophrenia↓  Schizophrenia ↑ | Whole blood  dEV plasma  sEV plasma | Jin et al. 2023  Chen et al. 2025 |
| miR-26a-5p | ↓ | Schizophrenia ↑ | Plasma | Shafiee-Kandjani et al. 2023 |
| miR-125a-5p | ↓ | - |  |  |
| miR-338-3p | ↓ | - |  |  |
| miR-1260a | ↓ | Recurrent depressive disorder ↓ | Plasma | Dobre et al. 2025 |
| miR-23b-3p | ↓ | Alzheimer’s disease ↑ | Plasma | Nagaraj et al. 2017 |
| miR-221-3p | ↓ | Major depressive disorder ↑ | Serum | Wan et al. 2015 |
| miR-27a-3p | ↓ | - |  |  |
| miR-30a-5p | ↓ | Schizophrenia ↓ | PMBC | Liu et al. 2017 |
| miR-205-5p | ↓ | Major depressive disorder ↑ | Serum  Plasma  PBMC | Morgunova et al. 2025 |
| miR-92b-3p | ↓ | Major depressive disorder ↓ | Plasma | Matei et al. 2025 |
| miR-197-3p | ↓ | Major depressive disorder ↑ | Plasma | Gecys et al. 2022 |
| miR-27b-3p | ↓ | - |  |  |
| miR-125b-5p | ↓ | Obsessive compulsive disorder ↓ | Plasma | Korkmaz et al. 2025 |
| miR-145-5p | ↓ | Alzheimer’s disease ↑ | Plasma | Wen et al. 2024 |
|  |  | Bipolar disorder ↓ | Whole blood | Tekin et al. 2022 |
| let-7d-5p | ↓ | Alzheimer’s disease ↑ | Serum | Poursaei et al. 2022 |
| miR-301a-3p | ↓ | Bipolar affective disorder ↓ | Plasma derived exosomes | Ceylan et al. 2020 |
| miR-29c-3p | ↓ | - |  |  |
| let-7e-5p | ↓ | Major depressive disorder ↑ | Plasma | Gecys et al. 2022 |
|  |  | Bipolar affective disorder ↑ | Plasma | Gecys et al. 2022 |
| miR-191-5p | ↓ | - |  |  |
| miR-16-5p | ↑ | - |  |  |
| miR-363-3p | ↑ | - |  |  |

miRNAs are ordered according to prior evidence: those previously investigated in psychotic disorders are listed first, followed by miRNAs reported in other psychiatric conditions.

dEV: extracellular vesicles depleted plasma

sEV: extracellular vesicles from plasma

**References**

- 44. Du X, Lv J, Feng J, Li X, Gao Y, Wang X, et al. Plasma exosomes lncRNA-miRNA-mRNA network construction and its diagnostic efficacy identification in first-episode schizophrenia. BMC Psychiatry. 2023 Dec 1;23(1). doi:10.1186/S12888-023-05052-9 PubMed PMID: 37605121.
- 45. Jin M, Liu Y, Hu G, Li X, Jia N, Cui X, et al. Establishment of a schizophrenia classifier based on peripheral blood signatures and investigation of pathogenic miRNA-mRNA regulation. J Psychiatr Res. 2023 Mar 1;159:172–84. doi:10.1016/J.JPSYCHIRES.2023.01.035 PubMed PMID: 36738648.
- 46. Chen BY, Lin JJ, Tseng HH, Huang CC, Chen PS, Li CH, et al. Profiling small extracellular vesicles microRNAs and their expressions in EVs-depleted plasma as biomarkers for distinguishing schizophrenia. Prog Neuropsychopharmacol Biol Psychiatry. 2025 Dec 20;143:111543. doi:10.1016/J.PNPBP.2025.111543 PubMed PMID: 41161522.
- 35. Shafiee-Kandjani AR, Nezhadettehad N, Farhang S, Bruggeman R, Shanebandi D, Hassanzadeh M, et al. MicroRNAs and pro-inflammatory cytokines as candidate biomarkers for recent-onset psychosis. BMC Psychiatry. 2023 Dec 1;23(1). doi:10.1186/S12888-023-05136-6 PubMed PMID: 37644489.
- 47. Dobre M, Manuc TE, Manuc M, Matei IC, Dobre AM, Dragne AD, et al. Circulating miRNA Profile in Inflammatory Bowel Disease Patients with Stress, Anxiety, and Depression. Int J Mol Sci. 2025 Aug 1;26(15):7321. doi:10.3390/IJMS26157321/S1 PubMed PMID: 40806453.
- 48. Nagaraj S, Laskowska-Kaszub K, Dębski KJ, Wojsiat J, Dąbrowski M, Gabryelewicz T, et al. Profile of 6 microRNA in blood plasma distinguish early stage Alzheimer’s disease patients from non-demented subjects. Oncotarget. 2017 Feb 5;8(10):16122–43. doi:10.18632/ONCOTARGET.15109 PubMed PMID: 28179587.
- 49. Wan Y, Liu Y, Wang X, Wu J, Liu K, Zhou J, et al. Identification of Differential MicroRNAs in Cerebrospinal Fluid and Serum of Patients with Major Depressive Disorder. PLoS One. 2015 Mar 12;10(3):e0121975. doi:10.1371/JOURNAL.PONE.0121975 PubMed PMID: 25763923.
- 50. Liu S, Zhang F, Shugart YY, Yang L, Li X, Liu Z, et al. The early growth response protein 1-miR-30a-5p-neurogenic differentiation factor 1 axis as a novel biomarker for schizophrenia diagnosis and treatment monitoring. Translational Psychiatry 2017 7:1. 2017 Jan 10;7(1):e998–e998. doi:10.1038/tp.2016.268 PubMed PMID: 28072411.
- 51. Morgunova A, O’Toole N, Abboud F, Coury S, Chen GG, Teixeira M, et al. Peripheral MicroRNA Signatures in Adolescent Depression. Biological Psychiatry Global Open Science. 2025 Jul 1;5(4):100505. doi:10.1016/J.BPSGOS.2025.100505 PubMed PMID: 40519638.
- 52. Matei IC, Milanesi E, Dobre M. Plasma-based microRNA biomarkers for depression in Romanian patients: preliminary findings. European Psychiatry. 2025 Apr;68(S1):S757–S757. doi:10.1192/J.EURPSY.2025.1535
- 37. Gecys D, Dambrauskiene K, Simonyte S, Patamsyte V, Vilkeviciute A, Musneckis A, et al. Circulating hsa-let-7e-5p and hsa-miR-125a-5p as Possible Biomarkers in the Diagnosis of Major Depression and Bipolar Disorders. Dis Markers. 2022;2022:3004338. doi:10.1155/2022/3004338 PubMed PMID: 35178127.
- 53. Korkmaz ND, Elibol B, Durmus Z, Ozdemir A, Akbas F, Guloksuz S, et al. Evaluation of potential biomarker miRNAs and the levels of serotonin and dopamine in female patients with obsessive–compulsive disorder. Neuroscience. 2025 Aug 16;581:50–7. doi:10.1016/J.NEUROSCIENCE.2025.07.001 PubMed PMID: 40614924.
- 54. Wen Q, Wittens MMJ, Engelborghs S, van Herwijnen MHM, Tsamou M, Roggen E, et al. Beyond CSF and Neuroimaging Assessment: Evaluating Plasma miR-145-5p as a Potential Biomarker for Mild Cognitive Impairment and Alzheimer’s Disease. ACS Chem Neurosci. 2024 Mar 6;15(5):1042–54. doi:10.1021/ACSCHEMNEURO.3C00740/ASSET/IMAGES/LARGE/CN3C00740_0006.JPEG PubMed PMID: 38407050.
- 55. Tekin SS, Erdal ME, Asoğlu M, Ay Öİ, Ay ME, Yılmaz SG. Biomarker potential of hsa-miR-145-5p in peripheral whole blood of manic bipolar I patients. Brazilian Journal of Psychiatry. 2022;44(4):378. doi:10.47626/1516-4446-2021-2260 PubMed PMID: 35749663.
- 56. Poursaei E, Abolghasemi M, Bornehdeli S, Shanehbandi D, Asadi M, Sadeghzadeh M, et al. Evaluation of hsa-let-7d-5p, hsa-let-7g-5p and hsa-miR-15b-5p plasma levels in patients with Alzheimer’s disease. Psychiatr Genet. 2022 Feb 1;32(1):25–9. doi:10.1097/YPG.0000000000000303 PubMed PMID: 34955516.
- 57. Ceylan D, Tufekci KU, Keskinoglu P, Genc S, Özerdem A. Circulating exosomal microRNAs in bipolar disorder. J Affect Disord. 2020 Feb 1;262:99–107. doi:10.1016/J.JAD.2019.10.038 PubMed PMID: 31726266.
- 37. Gecys D, Dambrauskiene K, Simonyte S, Patamsyte V, Vilkeviciute A, Musneckis A, et al. Circulating hsa-let-7e-5p and hsa-miR-125a-5p as Possible Biomarkers in the Diagnosis of Major Depression and Bipolar Disorders. Dis Markers. 2022;2022:3004338. doi:10.1155/2022/3004338 PubMed PMID: 35178127.
